# Myasthenia gravis and pregnancy: a systematic review and meta-analysis

**DOI:** 10.1007/s00415-026-13724-1

**Published:** 2026-03-05

**Authors:** Lisa Miegel, Julia Hickstein, Antonia Reibelt, Christoph Heesen, Charlotte Schubert

**Affiliations:** 1 Institute of Neuroimmunology and Multiple Sclerosis, University Medical Center Hamburg-Eppendorf, Hamburg, Germany; 2 Institute of Social Medicine and Epidemiology, University of Lübeck, Lübeck, Germany; 3 Department of Neurology, University Medical Center Hamburg-Eppendorf, Hamburg, Germany

**Keywords:** Myasthenia gravis, Pregnancy, Breast feeding, Systematic review

## Abstract

**Background:**

Myasthenia gravis (MG) is an autoimmune disease with an onset age distribution in women, which peaks within the reproductive age range, leading to a potential interference with pregnancy. In this systematic review, we aim to assess the impact of pregnancy on the course of MG during pregnancy and postpartum period and to identify potential risk factors influencing the postpartum course.

**Methods:**

A systematic literature search was conducted in the two databases PubMed and Epistemonikos on the topic “Myasthenia gravis and pregnancy” in October 2025. We included cohort studies and case series with at least 5 cases, reporting the clinical course of MG in women during pregnancy or postpartum period. Quality assessment was performed using the Critical Appraisal Skills Programme (CASP) tool for cohort studies.

**Results:**

In total, 34 studies were included, covering 3720 pregnancies in women with MG. Of 842 pregnancies worsening myasthenic symptoms was reported in 30% of pregnancies most frequently in the first trimester. Postpartum exacerbations were observed in 27% of the cases. However, incidence of worsening and scoring systems differed vastly. Preterm birth was reported at a rate of 9%. Vaginal delivery and cesarean section were performed in 68% and 32% respectively. Of 1530 infants, 9% developed neonatal myasthenia gravis.

**Conclusion:**

This review confirms that symptom worsening is most common in the first trimester or postpartum. Women with MG were more likely to require cesarean section or operative vaginal delivery compared to general population data. However, the studies’ quality varied widely. Prospective studies are needed to better identify risk factors for complicative course during pregnancy and postpartum period.

**Supplementary Information:**

The online version contains supplementary material available at 10.1007/s00415-026-13724-1.

## Introduction

Overlapping with the reproductive age in women, myasthenia gravis (MG) can potentially interfere with pregnancy in female patients [1–3]. Therefore, MG can not only impact pregnancy, but also the course of MG can potentially be influenced by physical and immunological adaptations during pregnancy. Characterized by fluctuating symptoms that increase under stress, MG patients might be more vulnerable to physical and psychological challenges during pregnancy and postpartum period [4]. The reports of the overall rate of worsening in MG patients during pregnancy cover a wide range, from 0% to over 50% [5, 6]. Also rates of pregnancy outcomes with regard to obstetric features [premature rupture of membranes (PROM), intrauterine growth restriction (IUGR), and small for gestational age (SGA)] and delivery (mode of delivery, preterm delivery) vary in literature. Notably, MG during pregnancy can not only influence the course of the disease in the mother but may also affect neonatal outcome. Autoantibodies that pass through the placenta can cause neonatal myasthenic syndrome in the infant, known as transient neonatal myasthenia gravis (TNMG). The present literature reports varying rate of TNMG in infants of mothers with MG from 2 to 33% [1, 7]. Regarding the postpartum period, previous studies have shown a beneficial effect of exclusive breastfeeding on relapse rates of other autoimmune diseases (AID) [8]. Moreover, other studies suggest an association of prolonged breastfeeding with decreased risk of postpartum onset of MG [9]. To the best of our knowledge, at present, the possible effect of breastfeeding on postpartum course of MG has not been systematically studied.

While the latest systematic review addressing the course of MG during pregnancy included studies up to 2020 [1] The present study aims to systematically review all reported case series of pregnancy in MG patients up to October 2025 to provide an evidence-based balanced understanding of the course of MG during pregnancy and postpartum period also during the changing landscape of immunotherapy in MG and potentially to identify new pregnancy-related risk factors that influence the course of MG postpartum.

## Methods

### Data sources and search strategy

**Fig. 1:**
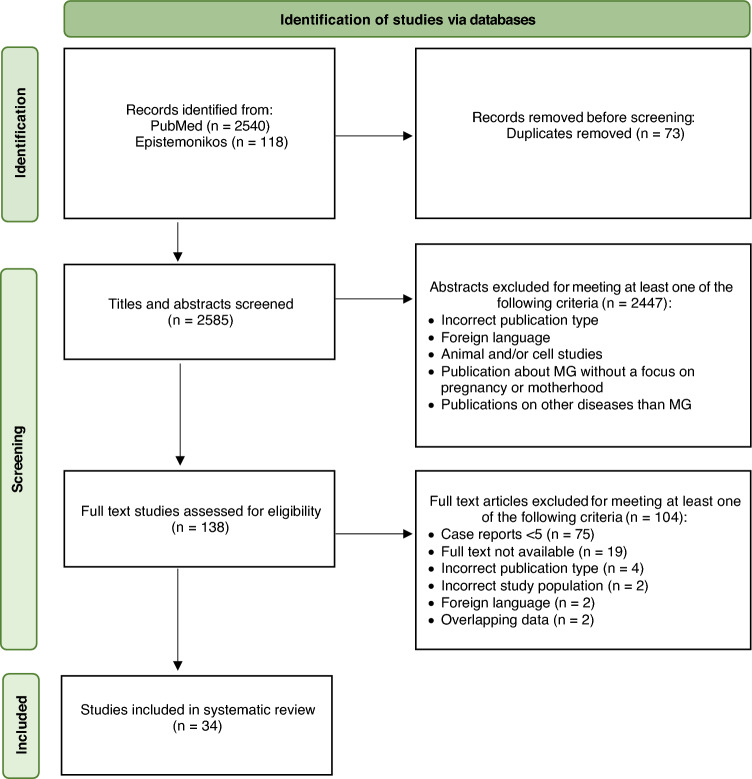
PRISMA (The Preferred Reporting Items for Systematic reviews and Meta-Analyses) flow diagram of study selection. Study identification, screening, quality assessment, and selection for this systematic review and meta-analysis

The methods of this systematic review were performed in accordance with the PRISMA (Preferred Reporting Items for Systematic Reviews and Meta-Analyses) guideline (Fig. 1). The electronic databases PubMed and Epistemonikos were systematically searched on the topic ‘Myasthenia gravis and pregnancy’. A comprehensive search strategy was developed for a scoping review and was used in this systematic review (Supplementary Tables 1 and 2). Broad search terms were used to maximize the sensitivity of the literature search and capture all potentially relevant studies concerning the pregnancy, postpartum and neonatal outcome in MG. This approach was chosen to account for variability in terminology across the literature and to minimize the risk of missing relevant studies. All databases were searched for results from inception up to 8th October 2025. Management and screening of all references were performed using EndNote citation management software and the systematic review software *Rayyan* [10]. After resolving duplicates, two authors (CS and LM) independently screened titles and abstracts of all references that resulted from the named search strategy. Studies were included when meeting the following criteria: (1) randomized-controlled trials, prospective/retrospective cohort studies, and case series, (2) MG diagnosis before, during, or within 6 months after pregnancy, (3) information on clinical MG status throughout pregnancy, and (4) publications written in English or German. Exclusion criteria included (1) commentaries or letters to the editor, (2) reviews about MG without a focus on pregnancy or motherhood, (3) publications on general risk factors of MG, (4) randomized-controlled trials with the specification ‘pregnant women are/were excluded’, (5) effects of pregnancy on the immune system without linkage to disease-specific data on MG, and (6) animal or cell studies.

Full-text screening was performed on the screened studies fulfilling the above-mentioned criteria, which were identified during title-abstract screening. LM and CS performed full-text screening independently. During full-text screening, studies were excluded meeting at least one of the following criteria: (1) publications with < 5 case reports, (2) full text not available, (3) incorrect publication type, (4) incorrect study population, or (5) full text in language other than English or German. Overlapping data among studies were resolved by excluding publications on previously reported data.

### Quality assessment

To assess the quality of cohort studies, two researchers independently used the Critical Appraisal Skills Programme (CASP) tool for cohort studies. During this process the included studies were rated in terms of the focus of the study, recruitment process, methodology for assessing exposure (pregnancy) and outcome (course of myasthenia gravis), identification of confounding factors, follow-up, precision estimates (CIs), and applicability of the results to the local population. Discrepancies were resolved by discussion. Highly selective recruitment and/or small cohort size (< 5 cases) led to the exclusion of the study from the review.

### Data extraction

Data extraction was performed after quality assessment using the CASP tool for cohort studies by LM. A standardized data extraction form was used to extract data from included studies. Extracted data included demographic information (age, parity, and comorbidities), disease information (time of diagnosis, duration of MG), disease features (serology, disease type, and thymectomy), treatment of disease (before, during pregnancy, and during postpartum period), obstetric features (PROM, IUGR, pre/term delivery, and mode of delivery), neonatal outcome (TNMG and SGA), and maternal outcome (course of disease during pregnancy and postpartum period in terms of worsenings, stable/unchanged disease, and improvements). The categories used in the present review were derived from those applied in the included studies. Owing to the limited methodological detail provided in these studies, a uniform and precise definition of the categories was difficult. If information was given, “worsening” was defined as a clinical aggravation of myasthenia gravis symptoms compared with the pre-pregnancy baseline, whereas “improvement” referred to a reduction in symptoms relative to the same baseline. Disease courses showing no relevant change were classified as “stable/unchanged”. In the studies included in the meta-analysis, “worsening” was defined according to the respective study-specific criteria, based on changes in MGFA classification or Osserman stage compared with baseline. Data were also extracted regarding the influence of breastfeeding on the course of MG postpartum, but only a few studies provided data on this matter.

### Statistical analysis

Descriptive statistics were performed using Excel (Version 16.41). Statistics on estimated probability of women with myasthenia gravis (wwMG) worsening during pregnancy was analyzed and visualized using R (R version 4.4.2; RStudio version 12.0). Temporal trends in treatment use were evaluated using weighted linear regression (weighted by study sample size), while pooled treatment proportions were estimated using random-effects meta-analysis of proportions (logit transformation) based on generalized linear mixed models; individual study estimates were visualized with bubble plots scaled by study size. Concerning the subanalysis of worsening during pregnancy in MG, for each study, the proportion of patients experiencing worsening during pregnancy was modeled as a binomial variable, and exact 95% confidence intervals were calculated based (Clopper-Pearson method). This was done by the binom.test function in R.

## Results

Our literature search resulted in 2658 references of which 73 duplicates were removed. Of the remaining 2585 references, 138 titles and abstracts were screened for full-text review. Ultimately, we identified 34 studies that fulfilled inclusion criteria and were rated eligible for our systematic review (Fig. 1).

**Fig. 2:**
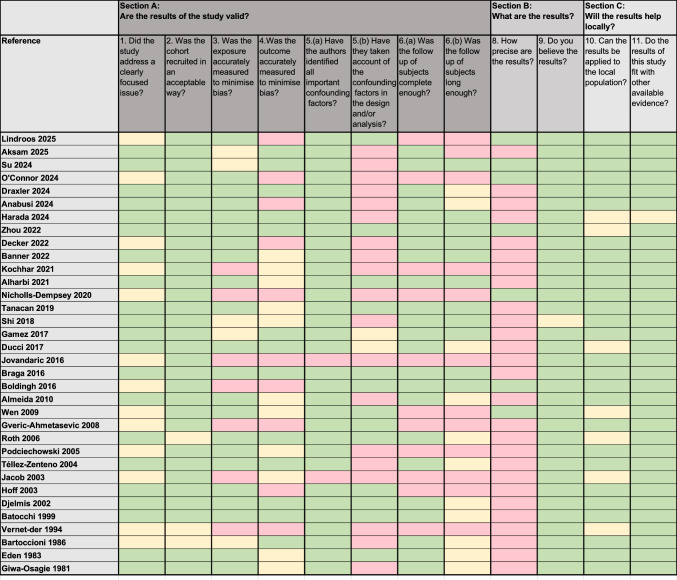
CASP (Critical Appraisal Skills Programme) quality assessment for cohort studies. Studies included in the systematic review. Quality assigned by color code according to 12 criteria: green = applicable; yellow = cannot tell; red = not applicable

Results of CASP quality assessment for cohort studies are summarized in Fig. 2. The overall risk of bias of the publications was high. Confounding factors were reported only in few publications. Confidence intervals were only described in 7 of 34 studies.

Baseline characteristics (demographic information, thymectomy, and treatment during pregnancy) from the 34 included studies are summarized in Supplementary Table 3. Included studies represent data from 21 different countries collected between 1981 up until 2025. Of the included publications, 21 were case series, 12 retrospective cohort studies, and 1 prospective cohort study. Sample size ranged between a minimum of 5 and a maximum of 974 included pregnancies in MG patients. In total, studies reported 3720 pregnancies in wwMG. The individual study design and outcome measurements of the included publications was highly variable, making comparative analyses between studies difficult.

### MG treatment in pregnancy

**Fig. 3:**
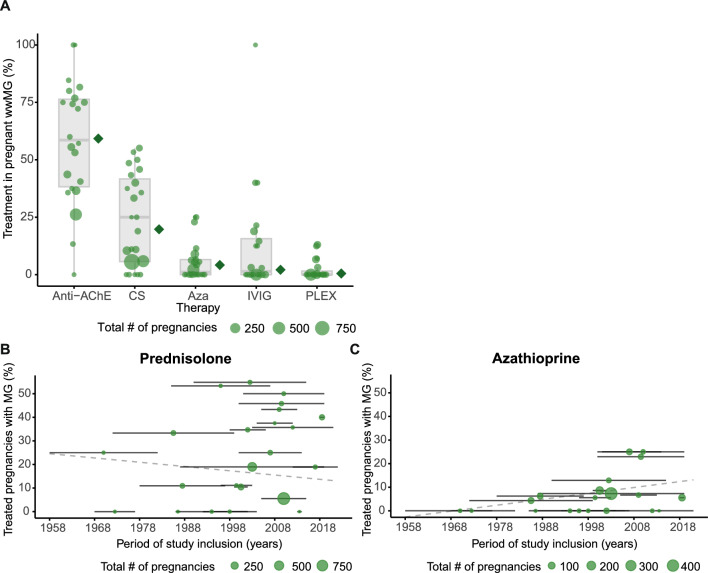
Use of immune modulating medication given during pregnancy period. Subanalysis of studies reporting treatment data. **A** Proportion of pregnancies exposed to each medication, expressed as percentage of total pregnancies per study. Bubble size reflects the total number of pregnancies in women with myasthenia gravis (wwMG) per study. The adjacent diamond indicates the pooled weighted estimate for each treatment. Temporal trends in the use of **B** prednisolone and **C** azathioprine according to the study inclusion period. Sample size-weighted linear regression is shown as a gray dashed line for prednisolone (*β* = − 0.18, 95% CI − 0.98 to 0.62, *p* = 0.64) and azathioprine (*β* = 0.25, 95% CI − 0.09 to 0.59, *p* = 0.14). *WwMG* women with myasthenia gravis, *Anti-AChE* anti-acetylcholine esterase inhibitors, *CS* corticosteroids, *Aza* azathioprine, *IVIG* i.v. immunoglobulins, *PLEX* plasmapheresis

The ratio of women who had a thymectomy before pregnancy ranged between 7 and 100% [7, 11]. Thymectomy during pregnancy or in postpartum period was reported in six studies ranging from 4% to over 50% with only 2 women reported as having thymectomy during pregnancy (Supplementary Table 3) [6, 12, 13]. During pregnancy, the majority of women were treated with anti-acetylcholinesterase (anti-AChE) (61%). 26 of 34 studies delivered additional information on the immunotherapy given (Fig. 3). A significant number of wwMG received corticosteroid (CS) therapy (16%) (Supplementary Table 3). Other MG specific medication during pregnancy comprised azathioprine (Aza), intravenous immunoglobulin (IVIG), and plasmapheresis (PLEX) (Fig. 3, Supplementary Table 3). Intravenous immunoglobulin and plasmapheresis were more frequently reported in recent studies and used in the context of myasthenic crises and exacerbations. Some studies also reported women who did not require any drug treatment at all trimesters during pregnancy with ranging proportions from 0 to 87% [5, 12, 14]. To provide a more detailed overview of treatment patterns, we analyzed the subset of 26 studies that reported additional information on MG medication. This analysis included overall treatment proportions (Fig. 3A) and changes in the frequency of prednisolone and azathioprine use over time. Weighted linear regression of prednisolone and azathioprine over the study inclusion period showed no significant temporal trend (Fig. 3B, C). Reported data on treatment of MG during pregnancy from the included studies are summarized in Supplementary Table 3.

### MG course in pregnancy and postpartum

During pregnancy, the proportion of women with exacerbations of MG ranges widely from 0 to 67% among studies [5, 14, 15]. In total, from 842 pregnancies, the course of MG was reported. Among these, 250 (30%) worsening events occurred during pregnancy. Of 11 studies with 452 pregnancies that reported the time point of exacerbation during pregnancy, the highest proportion occurred during the first trimester (20% of the 452). Among studies that also reported on improved MG course during pregnancy, proportions were generally lower compared to exacerbation rates, except for few publications [16–18]. The highest reported proportion of improved disease course among studies was 63% [18]. The observation of unchanged or stable disease course of MG during pregnancy ranged from 20% up to 89% [19, 20]. In total, unchanged, or stable disease course was reported in 52% among studies. One study reported the disease course during pregnancy as “improved or unchanged” without further differentiation and was therefore not included in the summary [21]. Myasthenic crises during pregnancy were infrequently reported with a highest rate of 11% and a total rate of 4% during pregnancy [22]. Some studies also included women with MG onset during pregnancy or postpartum period ($\le$ 6 months postpartum). Among studies, the highest number of cases with onset during pregnancy was reported with 24% with a total onset rate during pregnancy of 3% for all studies that reported time of onset [9].

Postpartum worsening rates ranged from 0 to 46% among studies [5, 23]. Highest reported improvement rate during postpartum period was 67% with a total improvement rate of 20% [14]. Only five studies reported the observation of unchanged/stable disease courses postpartum with a total proportion of 40%. One study reported disease course during postpartum period as “good/same” without further differentiation and was therefore not included in the summary [24]. For postpartum period myasthenic crises were infrequently reported in only eight studies with a highest rate of 20% and an average rate of 3% [13]. Reported data on MG course during pregnancy and postpartum period from the included studies are summarized in Supplementary Table 4.

Compared to pregnancy, total onset rate of MG during postpartum period over all studies was slightly higher with a rate of 4%. Highest reported proportion rate of MG onset during postpartum period was 76% [9]. In total, onset of MG during pregnancy or postpartum period was reported with a rate of 7%.

**Fig. 4:**
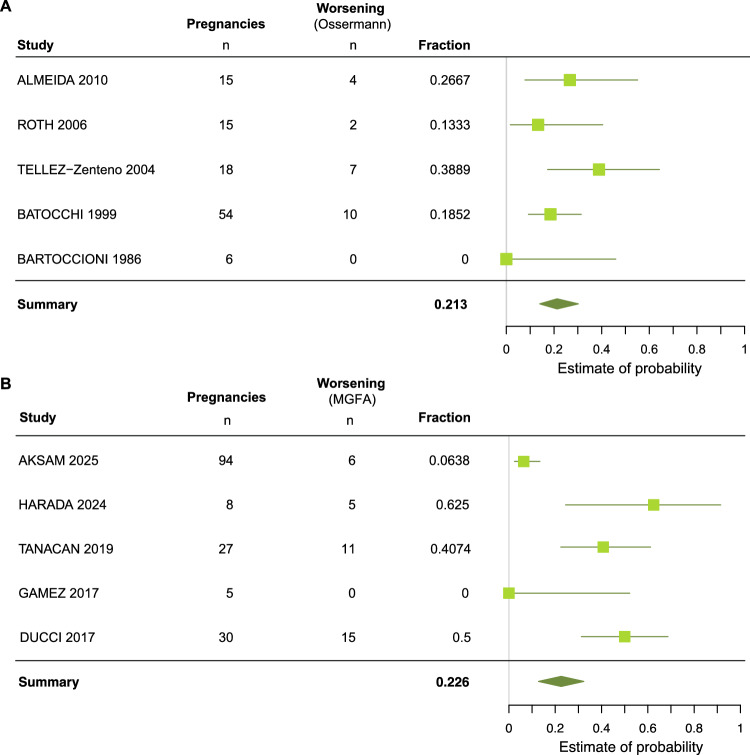
Subanalysis on reported estimated probability of MG worsening during pregnancy (all trimester) by weighted fraction of MG worsening measured by **A** Osserman classification and **B** MGFA classification

Notably, the underlying classification scores for assessment of MG course varied among studies. In subset of cohorts analyzed with the same assessment tool, we performed a meta-analysis on the estimated probability of worsening during 108 (Osserman criteria) and 164 (classification of the Myasthenia Gravis Foundation of America (MGFA) pregnancies which confirmed 21% and 23% of pregnancies to be affected during pregnancy with a substantial variability between studies (Fig. 4) [25, 26].

### Obstetric features and mode of delivery

Obstetric features were infrequently reported among studies. Hypertensive disorders (HDs) were reported in 13 studies with a total rate of 12%. Gestational diabetes mellitus (GDM) was reported at a rate total of 7% over all studies. Other obstetric complications such as premature rupture of membranes (PROM) were reported to occur with a total rate of 5%. In three studies that reported intrauterine growth retardations (IUGR), an overall rate of 2% was observed. Publications that reported postpartum hemorrhage (PPH) were similarly rare and reported a total rate 5%. Proportions of spontaneous abortion, miscarriage (MC), and perinatal death (PND) ranged widely in between studies with a total rate of approximately 2%. Regarding term and mode of delivery, outcomes were more frequently reported. Preterm birth before 37 weeks of pregnancy varied wide with a highest reported rate of 42% [7]. In total, preterm birth was reported with an overall rate of 9%. Vaginal delivery in contrast to cesarean section was more often performed with total rates of 68% and 32%.

### Neonatal outcome and breastfeeding postpartum

Of 1530 infants, 140 developed (transient) neonatal myasthenia gravis (9%). Other neonatal outcomes such as growth retardations (e.g., SGA) were infrequently reported with a total rate of 13%. Only a small number of the included studies reported cases of possible fetal acetylcholine receptor antibody-associated disorders (FARAD) such as the arthrogryposis multiplex congenita (AMC) in the newborns with a total rate of 0.4%. Breastfeeding during postpartum period was rarely reported. However, of 112 women, the majority initiated breastfeeding during the postpartum period without further specification about exclusivity (84%). Exclusiveness of breastfeeding, defined as breastfeeding for at least 2 months without regular replacement of any meal by supplemental feeding was reported in only one of the studies [20]. Reported data on obstetric features and neonatal outcome from the included studies are summarized in Supplementary Table 5.

## Discussion

This systematic review comprehensively summarizes the data on pregnancy and the postpartum period in wwMG by 34 studies with a total of 3720 pregnancies. The search strategy of our systematic review was designed broadly, aiming to capture all present studies on this issue to reflect the wide and heterogeneous group of myasthenia gravis patients. As a consequence of the broad acceptance criteria, the study design, the methods and the classifications of MG worsening varied among studies. Information on the degree and the duration of the clinical changes were missing in most of the cases; the overall quality of the included studies was moderate to low. Confidence intervals were reported only in a few cases, likely due to small sample sizes. In several publications, outcome measures were not clearly defined, confounding factors were not systematically addressed, and postpartum follow-up durations were not always clearly specified. These limitations should be considered when interpreting the findings.

In previous studies reporting individual case series, the range of exacerbation rates during pregnancy range wide from 0 to 66.6% [5, 14, 15]. The latest systematic review reported slightly higher rates of worsening of MG during pregnancy with 33.8% in comparison with our systematic review with predominance during the first trimester and a considerable worsening at the postpartum period [1]. A critical limitation of the available studies is the frequent absence of information on pre-pregnancy worsening rates, which hampers a robust assessment of baseline disease severity. In addition, the measurement instruments and definitions used to capture disease change vary substantially across studies, resulting in considerable methodological heterogeneity. We summarized the scores most often used—the Osserman classification and the MGFA—in a subgroup of studies published by a meta-analysis. This revealed even lower worsening rates of 21% and 23%. Given that this represents an exploratory subset analysis of 108 and 164 pregnancies, respectively, it should therefore not be interpreted as a severity-weighted or comprehensive probability of clinical worsening. Prospective, systematically designed studies are required to obtain a more reliable understanding of clinical trajectories during pregnancy and the postpartum period.

Regarding therapies, only a small proportion of women with MG received immunotherapy, despite increasing evidence supporting their overall safety [27]. No clear trends in therapy use over time were observed, and studies did not report outcomes stratified by treatment. Therefore, it is not possible to draw conclusions on the effects of specific therapies on maternal or neonatal outcomes.

Mode of delivery for pregnant women with MG already is much discussed. Since the uterus contains smooth muscle tissue, it is usually not affected by MG. Therefore, MG should not hinder the early stages of labor [28]. However, in the second stage of labor striated muscular effort is required during active pushing. In MG, patient’s fatigability and weakening might be exacerbated which leads to more frequent indications of assisted vaginal deliveries [28, 29]. Overall, in our systematic review, cesarean section was performed in 32% of births and 68% of births occurred by vaginal delivery. Regarding the indication for cesarean section, 98% were done for an obstetric indication and only 2% due to MG. Of vaginal deliveries, which were reported with further information regarding operative or spontaneous delivery, 88% of births occurred by spontaneous vaginal delivery (SVD) and 12% were operative vaginal delivery (OVD). In line with our observations, Hoff and his colleagues reported a significantly higher number of cesarean sections in an MG population compared to a reference group without MG [30]. This was confirmed in a Norwegian cohort recently [31]. However, percentages of caesarian sections vary worldwide [32]. Additionally, an increase in caesarian sections is reported in the last years [33]. Nevertheless, current guidelines advice women with MG to have spontaneous vaginal delivery and recommend that cesarean section should be reserved for obstetric indications [34].

In the general population, preterm delivery rates of life births ranged from 5.2 to 10.4% among different European countries in 2010 [35]. In Germany, a total rate of 8.4% was reported [35]. Summarizing the global region of Northern America, Australia and New Zealand, Central Asia, and Europe, 7.9 preterm births per 100 life births were reported in 2020 [36]. With 9%, the total rate of preterm births in MG patients of our systematic review was slightly higher. In addition, the previous studies observed an increased risk of PROM in MG patients compared to a 3% risk of the general population [1, 37]. In accordance with these studies, in our review, the overall rate of PROM was 5%.

Occurring shortly after birth, TNMG is the most common form of neonatal MG [38]. Previous literature reviews reported TNMG rates of 10% with a median for onset of 1 day and latest onset of 4 days after birth [38]. In line, in our systematic review, the total rate of neonatal MG was 9%. Therefore, infants of mothers with MG should be carefully observed for signs of myasthenic symptoms. Based on their observations, Kochhar and her colleagues suggest an observation period of 4 days for the clinician to follow infants born to mothers with MG [38].

At present, breastfeeding rates of mothers with MG are infrequently reported. In previous studies, breastfeeding was suggested to have protective effects on the course of AIDs after pregnancy [8]. Prior research even suggested an association of prolonged breastfeeding with decreased risk of postpartum onset of MG [9]. In publications that reported on breastfeeding initiation after pregnancy of women with MG, no adverse events were reported. However, the increased risk for breast inflammation as well as other puerperal infection can potentially worsen myasthenic syndromes and have to be taken into account [39]. For further evaluation of the effect of breastfeeding on the postpartum course of MG, more studies with bigger cohorts are needed and should be sought for in the future.

In this systematic approach, we confirm that worsenings are most likely to occur during the first trimester during pregnancy or after pregnancy during the first 6 months of postpartum period. In accordance with our findings regarding the course of MG in relation to pregnancy, rates of myasthenic crises were similar during pregnancy and postpartum period. In line with these findings, onset rates of MG during pregnancy and postpartum period were similar with slightly higher proportions during postpartum period. Among obstetric complications, hypertensive disorders in the mother and preterm birth were the most common in reviewed cohort. Women with MG were more likely to receive cesarean section or operative vaginal delivery compared to the general population. With regards to neonatal outcome, (T)NMG is a rare but in some cases severe consequence in infants. Neonates born to mothers with MG have an increased risk and should be carefully observed for signs of neonatal MG after birth.

A major limitation of this study is due to the inclusion of case series with varying study design. Naturally, the patient sample size in publications is usually small, reflective of the fact that MG is a rare neurological condition. Exclusion of studies reporting less than 5 cases was performed to minimize publications of extreme results.

## Conclusion

This large systematic review suggests that women with myasthenia gravis are at risk of symptom worsening, particularly in the first trimester and postpartum. However, most data are limited by heterogeneity in outcome definitions, reporting, and study design. Immunotherapies are only given in a minority of wwMG. Prospective studies are required to generate stronger evidence and optimize patient care in future.

## Supplementary Information

Below is the link to the electronic supplementary material.

Supplementary file1 (DOCX 111 KB)

## Data Availability

The data supporting the findings of this article are included within the main manuscript and in the supplementary material. Any additional data are available from the corresponding author upon reasonable request.

## References

[1] Banner H, Niles KM, Ryu M, Sermer M, Bril V, Murphy KE (2022) Myasthenia gravis in pregnancy: systematic review and case series. Obstet Med 15:108–117 35845224 10.1177/1753495X211041899 PMC9277733

[2] Grob D, Brunner N, Namba T, Pagala M (2008) Lifetime course of myasthenia gravis. Muscle Nerve 37:141–149 18059039 10.1002/mus.20950

[3] Su M, Liu X, Wang L et al (2022) Risk factors for pregnancy-related clinical outcome in myasthenia gravis: a systemic review and meta-analysis. Orphanet J Rare Dis 17:52 35172854 10.1186/s13023-022-02205-z PMC8848664

[4] Bogdan A, Barnett C, Ali A et al (2020) Chronic stress, depression and personality type in patients with myasthenia gravis. Eur J Neurol 27:204–209 31408565 10.1111/ene.14057

[5] Gamez J, Salvado M, Casellas M, Manrique S, Castillo F (2017) Intravenous immunoglobulin as monotherapy for myasthenia gravis during pregnancy. J Neurol Sci 383:118–122 29246598 10.1016/j.jns.2017.10.037

[6] Braga AC, Pinto C, Santos E, Braga J (2016) Myasthenia gravis in pregnancy: experience of a portuguese center. Muscle Nerve 54:715–720 26930188 10.1002/mus.25095

[7] Eden RD, Gall SA (1983) Myasthenia gravis and pregnancy: a reappraisal of thymectomy. Obstet Gynecol 62:328–333 6224102 10.1097/00006250-198309000-00013

[8] Schubert C, Steinberg L, Peper J et al (2023) Postpartum relapse risk in multiple sclerosis: a systematic review and meta-analysis. J Neurol Neurosurg Psychiatry 94:718–725 36807056 10.1136/jnnp-2022-330533

[9] Boldingh MI, Maniaol AH, Brunborg C, Weedon-Fekjær H, Verschuuren JJ, Tallaksen CM (2016) Increased risk for clinical onset of myasthenia gravis during the postpartum period. Neurology 87:2139–2145 27770065 10.1212/WNL.0000000000003339 PMC5109939

[10] Ouzzani M, Hammady H, Fedorowicz Z, Elmagarmid A (2016) Rayyan—a web and mobile app for systematic reviews. Syst Rev 5:210 27919275 10.1186/s13643-016-0384-4 PMC5139140

[11] Wen JC, Liu TC, Chen YH, Chen SF, Lin HC, Tsai WC (2009) No increased risk of adverse pregnancy outcomes for women with myasthenia gravis: a nationwide population-based study. Eur J Neurol 16:889–894 19486132 10.1111/j.1468-1331.2009.02689.x

[12] Roth TC, Raths J, Carboni G, Rösler K, Schmid RA (2006) Effect of pregnancy and birth on the course of myasthenia gravis before or after transsternal radical thymectomy. Eur J Cardiothorac Surg 29:231–235 16386922 10.1016/j.ejcts.2005.11.022

[13] Almeida C, Coutinho E, Moreira D, Santos E, Aguiar J (2010) Myasthenia gravis and pregnancy: anaesthetic management—a series of cases. Eur J Anaesthesiol 27:985–990 20733499 10.1097/EJA.0b013e32833e263f

[14] Giwa-Osagie OF, Newton JR, Larcher V (1981) Obstetric performance of patients with my asthenia gravis. Int J Gynaecol Obstet 19:267–270 6119253 10.1016/0020-7292(81)90073-4

[15] Podciechowski L, Brocka-Nitecka U, Dabrowska K, Bielak A, Hadacz B, Wilczynski J (2005) Pregnancy complicated by myasthenia gravis—twelve years experience. Neuro Endocrinol Lett 26:603–608 16264394

[16] Batocchi AP, Majolini L, Evoli A, Lino MM, Minisci C, Tonali P (1999) Course and treatment of myasthenia gravis during pregnancy. Neurology 52:447–452 10025772 10.1212/wnl.52.3.447

[17] Djelmis J, Sostarko M, Mayer D, Ivanisevic M (2002) Myasthenia gravis in pregnancy: report on 69 cases. Eur J Obstet Gynecol Reprod Biol 104:21–25 12128277 10.1016/s0301-2115(02)00051-9

[18] Harada Y, Bettin M, Juel VC et al (2023) Pregnancy in MuSK-positive myasthenia gravis: a single-center case series. Muscle Nerve 68:85–90 37150596 10.1002/mus.27839

[19] Ducci RD, Lorenzoni PJ, Kay CS, Werneck LC, Scola RH (2017) Clinical follow-up of pregnancy in myasthenia gravis patients. Neuromuscul Disord 27:352–357 28256306 10.1016/j.nmd.2017.01.021

[20] Zhou Q, Yin W, Zhu J et al (2022) Risk factors associated with adverse pregnancy outcomes and postpartum exacerbation in women with myasthenia gravis. Am J Reprod Immunol (New York NY 1989) 88:e13641 10.1111/aji.13641 36305609

[21] Anabusi S, Izenberg A, Barnett C, Berndl A (2024) Pregnancy planning may impact maternal and neonatal outcomes in people with myasthenia gravis. Muscle Nerve 69:318–324 38156425 10.1002/mus.28021

[22] Alharbi M, Menon D, Barnett C, Katzberg H, Sermer M, Bril V (2021) Myasthenia gravis and pregnancy: Toronto Specialty Center experience. Can J Neurol Sci 48:767–771 33431076 10.1017/cjn.2021.2

[23] Su M, Liu X, Wu Z et al (2024) Nomogram for predicting pregnancy-related relapse of myasthenia gravis. Orphanet J Rare Dis 19:452 39617942 10.1186/s13023-024-03466-6 PMC11610150

[24] Aksam S, Kocijancic Belojevic D, Dotlic J et al (2025) Pregnancy and neonatal outcomes in women with myasthenia gravis: two decades experience from a university clinic. Ginekol Pol 96:686–693 40418034 10.5603/gpl.102412

[25] Jaretzki A 3rd, Barohn RJ, Ernstoff RM et al (2000) Myasthenia gravis: recommendations for clinical research standards. Task Force of the Medical Scientific Advisory Board of the Myasthenia Gravis Foundation of America. Neurology 55:16–23 10891897 10.1212/wnl.55.1.16

[26] Osserman KE, Kornfeld P, Cohen E (1958) Studies in myasthenia gravis; review of two hundred eighty-two cases at the Mount Sinai Hospital, New York City. AMA Arch Intern Med 102:72–81 13558746 10.1001/archinte.1958.00260190074008

[27] Wiendl H, Abicht A, Chan A et al (2023) Guideline for the management of myasthenic syndromes. Ther Adv Neurol Disord 16:17562864231213240 38152089 10.1177/17562864231213240 PMC10752078

[28] Massey JM, De Jesus-Acosta C (2014) Pregnancy and myasthenia gravis. Continuum (Minneap Minn) 20:115–127 24492814 10.1212/01.CON.0000443840.33310.bd PMC10563915

[29] Varner M (2013) Myasthenia gravis and pregnancy. Clin Obstet Gynecol 56:372–381 23563874 10.1097/GRF.0b013e31828e92c0

[30] Hoff JM, Daltveit AK, Gilhus NE (2003) Myasthenia gravis: consequences for pregnancy, delivery, and the newborn. Neurology 61:1362–1366 14638956 10.1212/01.wnl.0000082725.21444.ec

[31] Lindroos JLV, Bjork MH, Cohen JM et al (2025) Obstetric and neonatal outcomes in patients with maternal myasthenia gravis: a nationwide cohort study. Neurology 105:e214139 41026991 10.1212/WNL.0000000000214139

[32] Boerma T, Ronsmans C, Melesse DY et al (2018) Global epidemiology of use of and disparities in caesarean sections. Lancet 392:1341–1348 30322584 10.1016/S0140-6736(18)31928-7

[33] Angolile CM, Max BL, Mushemba J, Mashauri HL (2023) Global increased cesarean section rates and public health implications: a call to action. Health Sci Rep 6:e1274 37216058 10.1002/hsr2.1274 PMC10196217

[34] Gerischer L, Meisel A (2024) Leitlinie für myasthene Syndrome (Myasthenia gravis, Lambert-Eaton-Myasthenie-Syndrom, Kongenitale myasthene Syndrome) für Patientinnen und Patienten. Deutsche Hirnstiftung (DHS), Deutsche Gesellschaft für Neurologie (DGN) und Deutsche Myasthenie Gesellschaft

[35] Delnord M, Blondel B, Zeitlin J (2015) What contributes to disparities in the preterm birth rate in European countries? Curr Opin Obstet Gynecol 27:133–142 25692506 10.1097/GCO.0000000000000156 PMC4352070

[36] Ohuma EO, Moller AB, Bradley E et al (2023) National, regional, and global estimates of preterm birth in 2020, with trends from 2010: a systematic analysis. Lancet 402:1261–1271 37805217 10.1016/S0140-6736(23)00878-4

[37] Smith G, Rafuse C, Anand N et al (2005) Prevalence, management, and outcomes of preterm prelabour rupture of the membranes of women in Canada. J Obstet Gynaecol Can 27:547–553 16100631 10.1016/s1701-2163(16)30711-3

[38] Kochhar P, Schumacher R, Sarkar S (2021) Transient neonatal myasthenia gravis: refining risk estimate for infants born to women with myasthenia gravis. J Perinatol 41:2279–2283 33597740 10.1038/s41372-021-00970-6

[39] Plauche WC (1964) Myasthenia gravis in pregnancy. Am J Obstet Gynecol 88:404–409 14123415

