# Supplementary material for: Myasthenia gravis and pregnancy: a systematic review and meta-analysis
Source: J Neurol. 2026 Mar 5;273(3):184. doi: 10.1007/s00415-026-13724-1 (PMC12963153; doi:10.1007/s00415-026-13724-1)
Supplement: Supplementary file 1 — Supplementary file1 (DOCX 111 KB) [file 415_2026_13724_MOESM1_ESM.docx]

**Myasthenia gravis and pregnancy: A systematic review and meta-analysis**

**Lisa Miegel^1^, Julia Hickstein^2^, Antonia Reibelt**^3^**, Christoph Heesen^1^**^†^**, Charlotte Schubert^1^**^†^

^1^Institute of Neuroimmunology and Multiple Sclerosis, University Medical Center Hamburg-Eppendorf, Hamburg, Germany

^2^Institute of Social Medicine and Epidemiology, University of Lübeck, Lübeck, Germany

^3^Department of Neurology, University Medical Center Hamburg-Eppendorf, Hamburg, Germany

†authors contributed equally

**Correspondence**: Charlotte Schubert, [cha.schubert@uke.de](mailto:cha.schubert@uke.de)

**Tables**

|  | Search strategy Pubmed |
| --- | --- |
| 1. | myasthenia |
| 2. | “myasthenia-gravis” |
| 3. | “myasthenia-gravis” |
| 4. | “myasthenia gravis”[MeSH Terms] |
| 5. | "Myasthenia Gravis, Autoimmune, Experimental"[Mesh Terms] |
| 6. | 1 or 2 or 3 or 4 or 5 |
| 7. | “pregnant woman” |
| 8. | “pregnant women” |
| 9. | „with child“ |
| 10. | pregnancy |
| 11. | pregnan* |
| 12. | gestation* |
| 13. | parturition |
| 14. | parturition* |
| 15. | motherhood |
| 16. | maternity |
| 17. | maternal |
| 18. | parity |
| 19. | gravidity |
| 20. | gravid* |
| 21. | nulliparity |
| 22. | nulligravid* |
| 23. | Primiparity |
| 24. | primigravid* |
| 25. | Multiparity |
| 26. | multigravid* |
| 27. | “family planning” |
| 28. | “desire to have children” |
| 29. | parenting |
| 30. | “week of pregnancy” |
| 31. | peripartal |
| 32. | “post partum” |
| 33. | post-partum |
| 34. | postpartum |
| 35. | “postpartum period” |
| 36. | labor |
| 37. | labour |
| 38. | “labor pain” |
| 39. | “labour pain” |
| 40. | childbirth* |
| 41. | birth* |
| 42. | delivery |
| 43. | “caesarean delivery” |
| 44. | section |
| 45. | “spontaneous vaginal birth” |
| 46. | abort* |
| 47. | puerperium |
| 48. | birth pangs” |
| 49. | contractions |
| 50. | throes |
| 51. | travail |
| 52. | “pregnant people”[MeSH Terms] |
| 53. | pregnancy[MeSH Terms] |
| 54. | Parturition[MeSH Terms] |
| 55. | “postpartum period”[MeSH Terms] |
| 56. | parenting[MeSH Terms] |
| 57. | “labor pain”[MeSH Terms] |
| 58. | parity[MeSH Terms] |
| 59. | gravidity[MeSH Terms] |
| 60. | “breast-feeding” |
| 61. | breastfeeding |
| 62. | breastfed |
| 63. | breastfeed |
| 64. | “nursing mothers” |
| 65. | “nursing women” |
| 66. | “nursing woman” |
| 67. | lactating |
| 68. | “breast feeding”[MeSH Terms] |
| 69. | 7 or 8 or 9 or 10 or 11or 12 or 13 or 14 or 15 or 16 or 17 or 18 or 19 or 20 or 21 or 22 or 23 or 24 or 25 or 26 or 27 or 28 or 29 or 30 or 31 or 32 or 33 or 34 or 35 or 36 or 37 or 38 or 39 or 40 or 41 or 42 or 43 or 44 or 45 or 46 or 47 or 48 or 49 or 50 or 51 or 52 or 53 or 54 or 55 or 56 or 57 or 58 or 59 or 60 or 61 or 62 or 63 or 64 or 65 or 66 or 67 or 68 |
| 70. | 6 and 69 |

**Supplementary Table 1 | Search Strategy Pubmed**.

Search on the topic “MG and pregnancy” with additional back- and forward reference tracking

|  | Search strategy Epistemonikos |
| --- | --- |
| 1. | myasthenia |
| 2. | “myasthenia-gravis” |
| 3. | “myasthenia-gravis” |
| 4. | 1 or 2 or 3 |
| 5. | “pregnant woman” |
| 6. | “pregnant women” |
| 7. | „with child“ |
| 8. | pregnancy |
| 9. | pregnan* |
| 10. | gestation* |
| 11. | parturition |
| 12. | parturition* |
| 13. | motherhood |
| 14. | maternity |
| 15. | maternal |
| 16. | parity |
| 17. | gravidity |
| 18. | gravid* |
| 19. | nulliparity |
| 20. | nulligravid* |
| 21. | Primiparity |
| 22. | primigravid* |
| 23. | Multiparity |
| 24. | multigravid* |
| 25. | “family planning” |
| 26. | “desire to have children” |
| 27. | parenting |
| 28. | “week of pregnancy” |
| 29. | peripartal |
| 30. | “post partum” |
| 31. | post-partum |
| 32. | postpartum |
| 33. | “postpartum period” |
| 34. | labor |
| 35. | labour |
| 36. | “labor pain” |
| 37. | “labour pain” |
| 38. | childbirth* |
| 39. | birth* |
| 40. | delivery |
| 41. | “caesarean delivery” |
| 42. | section |
| 43. | “spontaneous vaginal birth” |
| 44. | abort* |
| 45. | puerperium |
| 46. | birth pangs” |
| 47. | contractions |
| 48. | throes |
| 49. | travail |
| 50. | “breast-feeding” |
| 51. | breastfeeding |
| 52. | breastfed |
| 53. | breastfeed |
| 54. | “nursing mothers” |
| 55. | “nursing women” |
| 56. | “nursing woman” |
| 57. | lactating |
| 58. | 5 or 6 or 7 or 8 or 9 or 10 or 11or 12 or 13 or 14 or 15 or 16 or 17 or 18 or 19 or 20 or 21 or 22 or 23 or 24 or 25 or 26 or 27 or 28 or 29 or 30 or 31 or 32 or 33 or 34 or 35 or 36 or 37 or 38 or 39 or 40 or 41 or 42 or 43 or 44 or 45 or 46 or 47 or 48 or 49 or 50 or 51 or 52 or 53 or 54 or 55 or 56 or 57 |
| 59. | 4 and 58 |

**Supplementary Table 2 | Search Strategy Epistemonikos**.

Search on the topic “MG and pregnancy” with additional back- and forward reference tracking

| Study characteristics | | | | Study population | | Thymectomy | | Treatment during pregnancy | | | | | |
| --- | --- | --- | --- | --- | --- | --- | --- | --- | --- | --- | --- | --- | --- |
| Author Year | **Country** | **Study Era** | **Design** | **Total number of wwMG** | **Total number of pregnancies in wwMG** | **Before pregnancy** | **During/ after pregnancy** | **None** | **Anti-AChE** | **CS** | **Aza** | **IVIG** | **PLEX** |
| Lindroos 2025 | Norway | 1999-2022 | Retrospective cohort study | NA | 134 | 23/134 (17.16%) | NA | NA | 49/134 (36.57%) | 14/134 (10.45%) | 12/134 (8.96%) | ≤3/134^a^ (≤2.24%) | 0/134 (0%) |
| Aksam 2025 | Serbia | 2000-2020 | Retrospective cohort study | 94 | 94 | 79/94 (84.04%) | NA | 41/94 (43.62%) | 41/94 (43.62%) | NA | NA | NA | NA |
| Su 2024 | China | 2015-2021 | Retrospective cohort study | 113 | 113 | 12/113 (18.58%) | NA | NA | NA | NA | NA | NA | NA |
| O’Connor 2024 | Sweden | 1987-2019 | Retrospective cohort study | 289 | 443 | 224/443 (50.56%) | NA | NA | 116/137 (84.67%) | 26/137 (18.98%) | 10/137 (7.3%) | 0/137 (0%) | 0/137 (0%) |
| Draxler 2024 | Germany | 2008-2022 | Case series | 55 | 69 | 40/67 (59.70%) | NA | 20/69 (28.99%) | NA | NA | NA | NA | NA |
| Anabusi 2024 | USA | 2018 | Retrospective cohort study | 58 | 90 | NA | NA | 30/90 (33.33%) | 50/90 (55.56%) | 36/90 (40.00%) | 5/90 (5.56%) | 17/90 (18.89%) | 6/90 (6.67%) |
| Harada 2024 | USA | 2003-2021 | Case series | 10 | 14 | 7/10 (70.00%) | 1/10 (after) (10.00%) | 8/14 (57.14%) | 5/14 (35.71%) | 5/14 (35.71%) | 0/14 (0%) | 1/14 (7.14%) | 1/14 (7.14%) |
| Zhou 2022 | China | 2012-2022 | Case series | 33 | 37 | 8/37 (21.62%) | NA | NA | 15/37 (40.54%) | 7/37 (18.92%) | 0/37 (0%) | 0/37 (0%) | 0/37 (0%) |
| Decker 2022 | USA | 2015-2017 | Retrospective cohort study | 843 | 843 | NA | NA | NA | NA | NA | NA | NA | NA |
| Banner 2022 | Canada | 2000-2019 | Case series | 39 | 48 | 34/48 (70.83%) | NA | 9/48 (18.75%) | 36/48 (75%) | 22/48 (45.83%) | 11/48 (22.92%) | 7/48 (14.58%) | 6/48 (12.5%) |
| Kochhar 2021 | USA | 1996-2017 | Case series | 30 | 37 | 18/37 (48.65%) | NA | NA | NA | NA | NA | NA | NA |
| Alharbi 2021 | Canada | 2001-2019 | Case series | 20 | 28 | 15/20^b^ (75.00%) | NA | NA | NA | 14/28 (50.00%) | 7/28 (25.00%) | 6/28 (21.43%) | NA |
| Nicholls-Dempsey | USA | 2005-2015 | Retrospective cohort study | NA | 974 | NA | NA | NA | NA | 54/974 (5.5%) | NA | NA | NA |
| Tanacan 2019 | Turkey | 2010-2017 | Retrospective cohort study | 12 | 27 | 7/27 (25.93%) | NA | NA | NA | NA | NA | NA | NA |
| Shi 2018 | China | 2004-2012 | Case series | 8 | 8 | 5/8 (62.50%) | NA | 4/8 (50%) | 3/8 (37.5%) | 3/8 (37.5%) | 0/8 (0%) | 1/8 (12.5%) | 0/8 (0%) |
| Gamez 2017 | Spain | 2013-2014 | Case series | 5 | 5 | 5/5 (100%) | NA | 0/5 (0%) | 0/5 (0%) | 0/5 (0%) | 0/5 (0%) | 5/5 (100%) | 0/5 (0%) |
| Ducci 2017 | Brazil | 1990-2015 | Case series | 21 | 35 | 8/30 (26.67%) | NA | 4/31 (12.90%) | 26/31 (83.87%) | 17/31 (54.84%) | 4/31 (12.90%) | 1/31 (3.23%) | 0/31 (0%) |
| Jovandaric 2016 | Serbia | 2000-2014 | Case series | NA | 62 | NA | NA | NA | 4/4^c^ (100%) | 1/4^c^ (25.00%) | 1/4^c^ (25.00%) | NA | NA |
| Braga 2016 | Portugal | 2005-2013 | Case series | 25 | 30 | 18/25 (72.00%) | 1/25 (during) (4.00%) | 3/30 (10%) | 24/30 (80.00%) | 13/30 (43.30%) | 2/30 (6.67%) | 12/30 (40.00%) | 0/30 (0%) |
| Boldingh 2016 | Norway  Netherlands | 1950-2012  1952-2012 | Retrospective cohort study | 37 | 37 | 28/37^d^ (75.68%) | NA | NA | NA | NA | NA | NA | NA |
| Almeida 2010 | Portugal | 1985-2007 | Case series | 15 | 15 | NA | 1/15 (during) (6.67%) | 4/12^e^ (26.67) | 9/12^e^ (75.00%) | 8/12^e^ (66.67%) | 0/12^e^ (0%) | 6/12^e^ (50.00%) | 0/12^e^ (0%) |
| Wen 2009 | Taiwan | 2001-2003 | Retrospective cohort study | 163 | 163 | 11/163 (6.75%) | NA | NA | NA | NA | NA | NA | NA |
| Gveric-Ahmetasevic 2008 | Croatia | 1998-2006 | Prospective cohort study | 44 | 49 | 25/49 (51.02%) | NA | NA | 40/49 (81.63%) | 27/49 (55.10%) | 0/49 (0%) | 0/49 (0%) | NA |
| Roth 2006 | Switzerland | 1986-2002 | Retrospective cohort study | 6 | 15 | 7/15 (46.15%) | 8/15 (after) (53.30%) | 13/15 (86.67%) | 2/15 (13.33%) | 0/15 (0%) | 0/15 (0%) | 0/15 (0%) | 0/15 (0%) |
| Podciechowski 2005 | Poland | 1992-2004 | Case series | 13 | 13 | 6/13 (46.15%) | NA | 2/13 (15.38%) | 11/13 (84.62%) | 0/13 (0%) | 0/13 (0%) | 0/13 (0%) | 0/13 (0%) |
| Téllez-Zenteno 2004 | Mexico | 1996-2003 | Case series | 18 | 18 | 17/18 (94.44%) | NA | NA | 13/18 (72.22%) | 2/18 (11.11%) | 1/18 (5.55%) | 0/18 (0%) | 0/18 (0%) |
| Jacob 2003 | Oman | 1997-2000 | Case series | NA | 8 | NA | NA | NA | NA | NA | NA | 1/8 (12.5%) | NA |
| Hoff 2003 | Norway | 1967-2000 | Retrospective cohort study | 79 | 127 | 45/127 (35.43%) | NA | NA | NA | NA | NA | NA | NA |
| Djelmis 2002 | Croatia | 1972-1999 | Case series | 65 | 69 | 25/65 (38.46%) | NA | 16/69 (23.19%) | 53/69 (76.81%) | 23/69 (33.33%) | 3/69 (4.35%) | 0/69 (0%) | 9/69 (13.04%) |
| Batocchi 1999 | Italy | 1978-1997 | Case series | 47 | 64 | 42/47 (89.35%) | 2/47 (after) (4.26%) | 26/64 (40.63%) | 34/64 (53.13%) | 7/64 (10.94%) | 4/64 (6.25%) | 2/64 (3.13%) | 2/64 (3.13%) |
| Vernet-der Garabedian1994 | France | 1994 | Case series | 22 | 23 | 9/16 (56.25%) | NA | NA | NA | NA | NA | NA | NA |
| Bartoccioni 1986 | Italy | 1986 | Case series | 7 | 7 | NA | NA | 3/7 (42.86%) | 4/7 (57.14%) | 0/7 (0%) | 0/7 (0%) | 0/7 (0%) | 0/7 (0%) |
| Eden 1983 | USA | 1958-1982 | Case series | 8 | 12 | 12/12 (100%) | NA | 3/12 (25.00%) | 9/12 (75.00%) | 3/12 (25.00%) | 0/12 (0%) | 0/12 (0%) | 0/12 (0%) |
| Giwa-Osagie 1981 | England | 1968-1977 | Case series | 6 | 9 | 4/9^d^ (44.44%) | NA | 0/9 (0%) | 9/9 (100%) | 0/9 (0%) | 0/9 (0%) | 0/9 (0%) | 0/9 (0%) |
| Total |  |  |  | 2185 | 3720 | 743/1669 (44.51%)) | 13/112 (11.61%) | 186/590 (31.53%) | 553/900  (61.44%) | 282/1808 (15.6%) | 60/834 (7.19%) | 59-62/838 (7.04%-7.4%) | 24/753 (3.19%) |

**Supplementary Table 3 |** Study details of studies included in the systematic review. Anti-AChE, Anti-Acetylcholinesterase; Aza, Azathioprine; CS, Corticosteroid; IVIG, Intravenous Immunoglobuline; NA, Not available; PLEX, Plasmapheresis. ^a^ To protect the anonymity of individual participants authors did not disclose nonmissing cell counts between 1 and 3. ^b^ Thymectomies before delivery. ^c^ n=4, number mothers with newborns who had TNMG symptoms. ^d^ No information about time point of thymectomy. ^e^ n=12, number of women diagnosed with MG at partum.

|  | MG course during pregnancy | | | | | | | | MG course postpartum (delivery until $\boldsymbol{\leq}$ 6 months postpartum) | | | | | Assessment of MG course | |
| --- | --- | --- | --- | --- | --- | --- | --- | --- | --- | --- | --- | --- | --- | --- | --- |
| Author Year | **Worsened** | | |  | **Improved** | **Unchanged/ stable** | **Myasthenic crisis** | **Onset of MG** | **Worsening** | **Improved** | **Unchanged/ stable** | **Myasthenic crisis** | **Onset of MG** | **Ossermann Classification, MGFA, clinical assessment, Drug requirement** |  |
|  | **1. T** | **2. T** | **3. T** | **1.-3. T** |  |  |  |  |  |  |  |  |  |  |  |
| Lindroos 2025 | NA | NA | NA | NA | NA | NA | NA | NA | NA | NA | NA | NA | NA | NA |  |
| Aksam 2025 | NA | NA | NA | 6/94 (6.38%) | 30/94 (31.91%) | 58/94 (61.7%) | 0/94 (0%) | NA | 3/94 (3.19%) | 91/94 (96.81%)^h^ | | NA | NA | MGFA |  |
| Su 2024 | 52/113^a^ (46.02%) | 11/113^a^ (9.73%) | 0/113^a^ (0%) | 63/113^a^ (55.75%) | 18/113^a^ (15.93%) | 43/113^a^ (38.05%) | NA | 0/113^a^ (0%) | 52/113^a^ (46.02%) | 18/113 (15.93%) | 43/113 ^a^ (38.05%) | NA | 0/113 ^a^ (0%) | MG-ADL |  |
| O’Connor 2024 | NA | NA | NA | NA | NA | NA | NA | NA | NA | NA | NA | NA | NA | MG Diagnosis: ICD-10 code G70.0, ICD9 code 358.0 |  |
| Draxler 2024 | 21/63 (33.33%) | 10/63 (15.87) | 10/62 (16.12%) | 40/64 (62.5) | NA | 24/64 (37.50%) | NA | NA | 25/63 (39.68%) | NA | NA | NA | NA | QMG |  |
| Anabusi 2024 | NA | NA | NA | 17/90 (18.89%) | 61/90 (67.78%)^i^ | | NA | 0/90 (0%) | NA | NA | NA | NA | 0/90 (0%) | Patient survey: stable/ improved/ worsened |  |
| Harada 2024 | 2/8^b^ (25.00%) | 2/8^b^ (25.00%) | 1/8^b^ (0%) | 5/8^b^ (100%) | 0/8^b^ (62.50%) | NA | NA | 3/14 (21.43%) | 2/8^b^ (25.00%) | 2/8^b,c^ (25.00%) | NA | NA | 3/14 (21.43%) | MGFA |  |
| Zhou 2022 | 0/37 (0%) | 0/37 (0%) | 2/37 (5.41%) | 2/37 (5.41%) | 2/37 (5.41%) | 33/37 (89.19%) | NA | 0/37 (0%) | 10/36^d,e^ (27.78%) | NA | NA | NA | 0/37 (0%) | MG-ADL, SSQ |  |
| Decker 2022 | NA | NA | NA | NA | NA | NA | NA | NA | NA | NA | NA | NA | NA | MG Diagnosis: ICD-10 code G70.0X |  |
| Banner 2022 | NA | NA | NA | 12/48 (25%) | 3/48 (6.25%) | 33/48 (68.75%) | 4/48 (8.33%) | 2/48 (4.16%) | NA | NA | NA | 0/48 (0%) | 0/48 (0%) | Frequency/ severity of symptoms, drug requirement (dose), additional treatment modality |  |
| Kochhar 2021 | NA | NA | NA | 13/37 (35.14%) | NA | NA | NA | NA | NA | NA | NA | NA | NA | NA |  |
| Alharbi 2021 | NA | NA | NA | 7/28 (25.00%) | NA | NA | 3/28 (10.71%) | 2/28 (7.14%) | 9/28 (32.14%) | NA | NA | NA | 1/28 (3.57%) | Patient’s report, clinical assessment, varying MG scores (QMG, MG-QOL 15, MGII or SSQ) |  |
| Nicholls-Dempsey 2020 | NA | NA | NA | NA | NA | NA | NA | NA | NA | NA | NA | NA | NA | MG Diagnosis: ICD-9 code 358 |  |
| Tanacan 2019 | 4/27 (14.81%) | 5/27 (18.52%) | 2/27 (7.41%) | 11/27 (40.74%) | 7/27 (25.93%) | 9/27 (33.33%) | NA | 0/27 (0%) | 7/27 (25.93%) | 10/27 (37.04%) | NA | NA | 0/27 (0%) | MGFA Clinical Classification, MGFA post-intervention Status Classification, MGC Score, Drug requirement |  |
| Shi 2018 | NA | NA | NA | 2/8 (25.00%) | NA | 6/8 (75.00%) | NA | 0/8 (0%) | 1/8 (12.50%) | NA | NA | NA | 0/8 (0%) | NA |  |
| Gamez 2017 | 0/5 (0%) | 0/5 (0%) | 0/5 (0%) | 0/5 (0%) | 0/5 (0%) | 5/5 (100%) | 0/5 (0%) | 0/5 (0%) | 0/5 (0%) | 0/5 (0%) | 5/5 (100%) | 0/5 (0%) | 0/5 (0%) | MGFA, QMG |  |
| Ducci 2017 | NA | NA | NA | 15/30 (50.00%) | 9/30 (30.00%) | 6/30 (20.00%) | 2/30 (6.66%) | 1/30 (3.33%) | NA | NA | NA | 1/30 (3.33%) | 0/30 (0%) | MGFA, MGC, changes in drug requirement |  |
| Jovandaric 2016 | NA | NA | NA | NA | NA | NA | NA | NA | NA | NA | NA | NA | NA | NA |  |
| Braga 2016 | 6/30 (20%) | 0/30 (0%) | 7/30 (23.33%) | 13/30 (43.33%) | 1/30 (3.33%) | NA | 0/30 (0%) | 1/30 (3.33%) | 13/28^e^ (46.43%) | NA | NA | 1/30 (3.33%) | 0/30 (0%) | Exacerbations, development of new symptoms, drug requirement, MGFA |  |
| Boldingh 2016 | NA | NA | NA | NA | NA | NA | NA | 9/37 (24.32%) | NA | NA | NA | NA | 28/37 (75.68%) | MG Diagnosis: ICD-9 code 358.8, ICD-10 code G70.0 |  |
| Almeida 2010 | 0/15 (0%) | 3/15 (20%) | 1/15 (6.67%) | 4/15 (26.67%) | 3/15 (20.00%) | 8/15 (53.33%) | 0/15 (0%) | 0/15 (0%) | NA | NA | NA | 3/15 (20%) | 3/15 (20%) | Ossermann Classification |  |
| Wen 2009 | NA | NA | NA | NA | NA | NA | NA | 0/136 (0%) | NA | NA | NA | NA | 0/136 (0%) | MG Diagnosis: ICD-9- CM code 358.0 |  |
| Gveric-Ahmetasevic 2008 | NA | NA | NA | NA | NA | NA | NA | 0/49 (0%) | NA | NA | NA | NA | 0/49 (0%) | NA |  |
| Roth 2006 | NA | NA | NA | 2/15 (13.33%) | 1/15 (6.67%) | 12/15 (80.00%) | 0/15 (0%) | NA | 2/15 (13.33%) | 3/15 (20.00%) | 2/15 (13.33%) | 0/15 (0%) | NA | Ossermann Classification |  |
| Podcie-chowski 2005 | 0/13 (0%) | 0/13 (0%) | 0/13 (0%) | 0/13 (0%) | NA | NA | NA | 0/13 (0%) | NA | NA | NA | NA | 0/13 (0%) | NA |  |
| Téllez-Zenteno 2004 | 1/18 (5.56%) | 6/18 (33.3%) | 0/18 (0%) | 7/18 (38.89%) | 2/18 (11.11%) | 9/18 (50.00%) | 1/18 (5.56%) | 0/18 (0%) | NA | NA | NA | NA | 0/18 (0%) | Ossermann Classification, drug requirement |  |
| Jacob 2003 | NA | NA | NA | 1/18 (5.56%) | NA | NA | NA | 0/18 (0%) | NA | NA | NA | NA | 0/18 (0%) | MG Diagnosis: clinical picture, repetitive nerve stimulation tests, edrophonium test. |  |
| Hoff 2003 | NA | NA | NA | NA | NA | NA | NA | NA | NA | NA | NA | NA | NA | NA |  |
| Djelmis 2002 | 0/69 (0%) | 0/69 (0%) | 10/69 (14.49%) | 10/69 (14.49%) | 17/69 (24.64%) | 31/69 (44.93%) | NA | 9/65 (13.85%) | 11/69 (15.94%) | NA | NA | 1/69 (1.45%) | 0/65 (0%) | NA |  |
| Batocchi 1999 | 6/54 (11.1%) | 1/54 (1.85%) | 3/54 (5.56%) | 10/54 (18.52%) | 12/54 (22.22%) | 32/54 (59.3%) | 2/47^g^ (4.26%) | 0/54 (0%) | 15/54 (27.78%) | 7/54 (12.96%) | NA | NA | 0/54 (0%) | Osserman and Genkins |  |
| Vernet-der Garabedian 1994 | NA | NA | NA | NA | NA | NA | NA | NA | NA | NA | NA | NA | NA | Clinical, pharmacological, electromyographical, and immunological (anti-AChR antibodies) criteria |  |
| Bartoccioni 1986 | NA | NA | NA | NA | NA | 6/7 (85.71%) | NA | 1/7 (14.29%) | NA | NA | 6/7 (85.71%) | NA | 0/7 (0%) | Ossermann Classification |  |
| Eden 1983 | NA | NA | NA | 4/12 (33.33%) | 3/12 (25%) | 5/12 (41.67%) | 1/12 (8.33%) | 0/12 (0%) | 1/12 (8.33%) | NA | NA | 1/12 (8.33%) | 0/12 (0%) | NA |  |
| Giwa-Osagie 1981 | NA | NA | NA | 6/9 (66.66%) | 1/9 (11.11%) | 2/9 (22.22%) | NA | 0/9 (0%) | 0/9 (0%) | 6/9 (66.67%) | 3/9 (33.33%) | NA | 0/9 (0%) | Drug requirement |  |
| Total | 92/452 (20.35%) | 38/452 (8.41%) | 36/451 (7.98%) | 250/842  (29.69%) | 109/584 (18.66%) | 322/625 (51.52%) | 13/342 (3.8%) | 28/863 (3.24%) | 151/569 (26.54%) | 46/231 (19.91%) | 59/149 (39.60%) | 7/224 (3.13%) | 35/863 (4.06%) |  |  |

**Supplementary Table 4 |** MG course during and after pregnancy. Abbreviations: Anti-AChR, Anti-Acetylcholine receptor; ICD, International Classification of Diseases; MG, Myasthenia gravis; MG-ADL, Myasthenia Gravis Activities of Daily Living; MGC, Myasthenia Gravis Composite score; MGFA, Myasthenia Gravis Foundation of America Classification; MG-QOL 15, Myasthenia Gravis Quality of Life 15; NA, Not available; QMG, Quantitative Myasthenia Gravis score; SSQ, single simple question.
^a^ n=113, total number of women. ^b^ n=8, number of pregnancies with MG onset before pregnancy. ^c^ After treatment with IVIG or PLEX. ^d^ n=36, number of pregnancies. One patient was excluded from analysis by the authors. ^e^ n=28, number of full-term pregnancies. ^f^ n=58, number of first-time mothers included in the study. ^g^ Two women experienced several myasthenic crises. ^h^ Data were reported by the authors as “good/ same” and were not included in the summary. ^i^ Data were reported by the authors as “improved or unchanged symptoms” and were not included in the summary.

|  | Obstetric features | | | | | | | Mode of delivery | | | | | Neonatal outcome | |  | | Breastfeeding | |
| --- | --- | --- | --- | --- | --- | --- | --- | --- | --- | --- | --- | --- | --- | --- | --- | --- | --- | --- |
| Author Year | **HDs** | **GDM** | **PROM** | **IUGR** | **PTB**  **(<37 w)** | **PPH** | **MC, PND** | **SVD** | **OVD** | **TVD** | **CS** | | **TNMG/ NMG** | **SGA** | | **AMC/**  **FARIS** | **Yes** | **No** |
|  |  |  |  |  |  |  |  |  |  |  | **CS: Obstetric indication** | **CS: MG as Indication** |  |  | |  |  |  |
| Lindroos 2025 | 8/134 (5.97%) | 4/134 (2.98%%) | 19/134 (14.18%) | NA | 11/134 (8.21%) | NA | ≤3/134 (≤2.24%)^j^ | NA | 14/119 (11.76%) | 107/134 (79.85%) | 27/134 (20.15%) | NA | 5/134 (3.73%) | 16/132 (12.12%) | | 0/134 (0%) | NA | NA |
| Aksam 2025 | NA | NA | NA | NA | NA | NA | NA | NA | NA | 59/94 (62.77%) | 35/94 (37.23%) | NA | 12/94 (12.77%) | NA | | NA | NA | NA |
| Su 2024 | NA | NA | NA | NA | 3/113 (2.65%) | NA | 4/113^a^ (3.54%) | NA | NA | 29/79^b^ (36.71%) | 50/79^b^ (63.29%) | 0/79 (0%) | NA | NA | | NA | NA | NA |
| O’Connor 2024 | 9/443 (2.03%) | 10/443 (2.26%) | 6/443 (1.35%) | NA | 29/443 (6.55%) | 23/443 (5.19%) | NA | 313/443 (70.65%) | 44/443  (9.93%) | 357/443 (80.59%) | 88/443 (19.86%) | 0/443 (0%) | 12/443^c^ (2.71%) | NA | | 0/443 (0%) | NA | NA |
| Draxler 2024 | NA | NA | NA | NA | 7/54 (12.96%) | NA | NA | 28/60 (46.67%) | 9/60 (15.00%) | NA | 23/60 (38.30%) | NA | 2/58 (3.45%) | 9/55 (16.3%) | | 2/58 (3.45%) | NA | NA |
| Anabusi 2024 | 13/90 (14.44%) | NA | NA | NA | 8/67 (11.94%) | NA | NA | NA | NA | 41/67 (61.19%) | 26/67 (38.81%) | 0/67 (0%) | 2/67 (2.99%) | NA | | NA | NA | NA |
| Harada 2024 | 0/14 (0%) | 0/14 (0%) | NA | NA | NA | NA | 0/14 (0%) | NA | NA | 9/14 (64.29%) | 5/14 (35.17%) | 0/14 (0%) | 0/14^g^ (0%) | NA | | 0/14 (0%) | NA | NA |
| Zhou 2022 | 2/37 (5.41%) | 11/37 (29.73%) | 7/37 (18.92%) | NA | 2/37 (5.41%) | 1/37 (2.71%) | 0/37 (0%) | 13/37 (35.14%) | 4/37 (10.81%) | 17/37 (45.95%) | 20/37 (54.05%) | 0/37 (0%) | 9/37^g^ (24.32%) | NA | | 0/37 (0%) | E: 11/36 (30.56%)^d^  NE: 25/36^g^ (69.44%)^d^ | 0/36 (0%) |
| Decker 2022 | 187/843^e^ (22.18%) | 65/843 (7.71%) | 75/843 (8.90%) | NA | 59/843 (6.999%) | 58/843 (6.88%) | NA | NA | NA | NA | NA | NA | NA | NA | | NA | NA | NA |
| Banner 2022 | NA | NA | 4/48 (8.33%) | NA | 9/48 (18.75%) | NA | NA | 22/48 (45.83%) | 10/48 (20.83%) | 32/48 (66.67%) | 16/48 (33.33%) | 0/48 (0%) | 1/48 (2.08%) | 6/48 (12.5%) | | NA | NA | NA |
| Kochhar 2021 | NA | NA | NA | NA | NA | NA | NA | NA | NA | NA | NA | NA | 8/37 (21.62%) | NA | | NA | NA | NA |
| Alharbi 2021 | NA | NA | 0/28 (0%) | NA | 2/28 (7.14%) | NA | NA | NA | NA | 20/28 (71.43%) | 8/28 (28.57%) | 0/28 (0%) | 2/28 (7.14%) | NA | | 0/28 (0%) | NA | NA |
| Nicholls-Dempsey 2020 | 105/974 (10.78%) | 83/974 (8.52%) | 5/974 (0.51%) | 22/974 (2.26%) | 100/974 (10.27%) | 21/974 (2.16%) | 7/974^e^ (0.72%) | 615/974 (63.14%) | 47/974 (4.83%) | 662/974 (67.97%) | 312/974 (32.03%) | 0/974 (0%) | NA | NA | | 3/947 (0.31%) | NA | NA |
| Tanacan 2019 | NA | NA | 4/23 (17.39%) | NA | 3/23 (13.04%) | NA | 4/27 (14.81%) | NA | NA | 5/23 (21.74%) | 18/23 (78.06%) | 0/23 (0%) | 6/23^g^ (26.09%) | NA | | NA | IR: 18/23 (78.26%)^f^  MR: 11/18^h^ (47.83%)^f^ | 5/23 (21.74%) |
| Shi 2018 | NA | NA | NA | NA | 1/8 (12.50%) | NA | NA | NA | NA | 5/8 (62.50%) | 2/8 (25.00%) | 1/8 (12.50%) | 1/8 (12.50%) | NA | | 0/8 (0%) | NA | NA |
| Gamez 2017 | NA | NA | NA | NA | 0/5 (0%) | NA | NA | NA | NA | 2/5 (40.00%) | 3/5 (60.00%) | 0/5 (0%) | 0/5 (0%) | NA | | 0/5 (0%) | NA | NA |
| Ducci 2017 | 1/35^d^ (2.86%) | 2/35 (5.71%) | 7/35 (20.00%) | NA | 7/30 (23.33%) | NA | 4/35^e^ (11.43%) | 8/30 (26.67%) | 2/30 (6.67%) | 10/30 (33.33%) | 15/30 (50.00%) | 5/30 (16.67%) | 4/31^g^ (12.90%) | NA | | 0/31 (0%) | NA | NA |
| Jovandaric 2016 | NA | NA | NA | NA | NA | NA | NA | NA | NA | 32/62 (51.61%) | 30/62 (48.39%) | 0/62 (0%) | 4/62 (6.45%) | NA | | 0/62 (0%) | NA | NA |
| Braga 2016 | 0/30 (0%) | 7/30 (23.33%) | NA | 0/30 (0%) | 0/28 (0%) | NA | 2/30 (6.67%) | 5/28 (17.86%) | 5/28 (17.86%) | 10/28 (35.71%) | 8/28 (28.57%) | 10/28 (35.71%) | 2/28^g^ (7.14%) | NA | | 0/28 (0%) | NA | NA |
| Boldingh 2016 | NA | NA | NA | NA | NA | NA | NA | NA | NA | NA | NA | NA | NA | NA | | NA | NA | NA |
| Almeida 2010 | 1/15^d^ (6.67%) | 6/15 (40.00%) | 2/15 (13.33%) | NA | 2/15 (13.33%) | NA | 2/17 (11.76%) | NA | NA | 5/15 (33.33%) | 7/15 (46.67%) | 3/15 (20.00%) | 0/15^g^ (0%) | NA | | NA | NA | NA |
| Wen 2009 | 9/163^d^ (5.52%) | 6/163 (3.68%) | NA | NA | 13/163 (7.98%) | NA | NA | 81/163 (49.69%) | 9/163 (5.52%) | 90/163 (55.21%) | 73/163 (44.79%) | 0/163 (0%) | NA | 29/163 (17.79%) | | NA | NA | NA |
| Gveric-Ahmetasevic 2008 | NA | NA | NA | NA | NA | NA | NA | NA | NA | NA | NA | NA | 16/49 (32.65%) | NA | | 0/49 (0%) | NA | NA |
| Roth 2006 | NA | NA | NA | NA | NA | NA | 3/15 (20.00%) | 8/12 (66.67%) | 3/12 (25.00%) | 11/12 (91.67%) | 1/12 (8.33%) | 0/12 (0%) | 2/12^g^ (16.67%) | NA | | 0/12 (0%) | 12/12 (100%) | 0/12 (0%) |
| Podciechowski 2005 | NA | NA | NA | 1/14 (7.14%) | 1/14 (7.14%) | NA | NA | 6/13 (46.15%) | 1/13 (7.69%) | 7/13 (53.85%) | 6/13 (46.15%) | 0/13 (0%) | 1/14 (7.14%) | 1/14 (7.14%) | | 0/14 (0%) | NA | NA |
| Téllez-Zenteno 2004 | 2/18 (11.11%) | NA | NA | NA | NA | NA | 1/18 (5.56%) | 9/18 (50%) | 1/18 (5.56%) | 10/18 (55.56%) | 8/18 (44.44%) | 0/18 (0%) | 1/17^g^ (5.88%) | NA | | 0/17 (0%) | NA | NA |
| Jacob 2003 | NA | NA | NA | NA | NA | NA | NA | NA | NA | NA | NA | NA | 0/8 (0%) | 0/8 (0%) | | 0/8 (0%) | NA | NA |
| Hoff 2003 | NA | NA | 7/126 (5.56%) | NA | NA | 7/126 (5.56%) | 3/127 (2.36%) | 94/127 (74.01%) | 11/127 (8.66%) | 105/127 (82.68%) | 22/127 (17.32%) | 0/127 (0%) | 5/124^g^ (4.03%) | NA | | 3/124 (2.42%) | NA | NA |
| Djelmis 2002 | NA | NA | NA | NA | 5/69 (7.25%) | NA | 1/69 (1.45%) | 51/69 (73.91%) | 6/69 (8.70%) | 57/69 (82.61) | 11/69 (15.94%) | 1/69 (1.45%) | 21/70^g^ (30.00%) | 3/70 (4.29%) | | 0/70 (0%) | 25/33 (75.76%) | 8/33 (24.24%) |
| Batocchi 1999 | NA | NA | NA | NA | 4/54 (7.41%) | NA | 4/64^h^ (6.25%) | NA | NA | 38/54 (70.37%) | 16/54 (29.63%) | 0/54 (0%) | 5/55^g^ (9.09%) | NA | | 0/55 (0%) | NA | NA |
| Vernet-der Garabedian1994 | NA | NA | NA | NA | NA | NA | NA | NA | NA | NA | NA | NA | 12/23 (52.17%) | NA | | 0/23 (0%) | NA | NA |
| Bartoccioni 1986 | NA | NA | NA | NA | NA | NA | NA | NA | NA | NA | NA | NA | 3/7 (42.86%) | NA | | 0/7 (0%) | NA | NA |
| Eden 1983 | 3/12 (25.00%) | NA | NA | NA | 5/12 (41.67%) | NA | 2/12 (16.67%) | 8/12 (66.67%) | 2/12 (16.67%) | 10/12 (83.33%) | 2/12 (16.67%) | 0/12 (0%) | 3/10^g,i^ (33.33%) | NA | | NA | NA | NA |
| Giwa-Osagie 1981 | NA | NA | NA | NA | 1/9 (11.11%) | NA | 1/9 (11.11%) | 2/8 (25.00%) | 6/8 (75.00%) | 8/8 (100%) | 0/8 (0%) | 0/8 (0%) | 1/9^g^ (11.11%) | NA | | 0/9 (0%) | 3/8 (37.50%) | 5/8 (62.50%) |
| Total | 340/2808 (12.11%) | 194/2688 (7.22%) | 136/2706 (5.03%) | 23/1018 (2.26%) | 272/3171 (8.58%) | 110/2423 (4.54%) | 39-41/1695 (2.3-2.42%) | 1263/ 2042 (61.85%) | 174/2161 (8.05%) | 1738/2565 (67.76%) | 832/2625 (31.70%) | 20/2337 (0.86%) | 140/1530 (9.15%) | 64/490 (13.06%) | | 8/2183 (0.37%) | 94/112  (84%) | 18/112 (16.07%) |

**Supplementary Table 5 |** Obstetric features and neonatal outcomes. Abbreviations: AMC, Arthrogryposis multiplex congenita; BF, Breastfeeding; CS, Cesarean section; E, Exclusive; FARIS, Clinical descriptions suggestive for Fetal acetylcholine receptor inactivation syndrome; GDM, gestational diabetes mellitus; HDs, Hypertensive disorders (including chronic hypertension, gestational hypertension, preeclampsia, eclampsia, proteinuria and preeclampsia superimposed on chronic hypertension); IUGR, Intrauterine growth retardation; IR, Initiation rate; MC, Miscarriage; MG: Myasthenia gravis; MR, Maintenance rate; NA, Not available; NE, Nonexclusive; NMG, Neonatal myasthenia gravis; OVD, Operational vaginal delivery; PND, Perinatal death; PPH, Postpartum hemorrhage PROM, Premature rupture of membranes; PTB, Preterm birth (<37 weeks); SGA, Small for gestational age; SVD, Spontaneous vaginal delivery; TVD, Total vaginal delivery; TNMG, Transient neonatal myasthenia gravis. ^a^ Additionally 27 elective terminations reported. ^b^ n=79, number of full-term births. ^c^ Including ICD-10 codes P94.0 (transient neonatal MG), G70.0 (MG), G702 (congenital myasthenia), and ICD-9 codes 775.2 (neonatal MG) and 358A (MG). ^d^ Exclusive (E) and nonexclusive (NE) breastfeeding. ^e^ Including all intrauterine fetal deaths. ^f^ Breastfeeding initiation (IR) rate and breastfeeding maintenance rate (MR) at 6 months postpartum. ^g^ n=total number of life births, without PNDs and MCs. ^h^ Additionally one ectopic pregnancy and six voluntarily induced abortions were reported. ^i^ One infant, who died during neonatal period had (T)NMG. ^j^ To protect the anonymity of individual participants authors did not disclose nonmissing cell counts between 1 and 3.
